# Supplementary material for: DGCR8 haploinsufficiency leads to primate-specific RNA dysregulation and pluripotency defects
Source: Nucleic Acids Res. Author manuscript; Available in PMC 2025 Mar 26. (PMC11941479; doi:10.1093/nar/gkaf197)

Supplementary Data statement

Supplementary Data are available at NAR online. Uncropped western blot images are shown in Supplementary Figure S8-S10.

Supplementary Figure S1

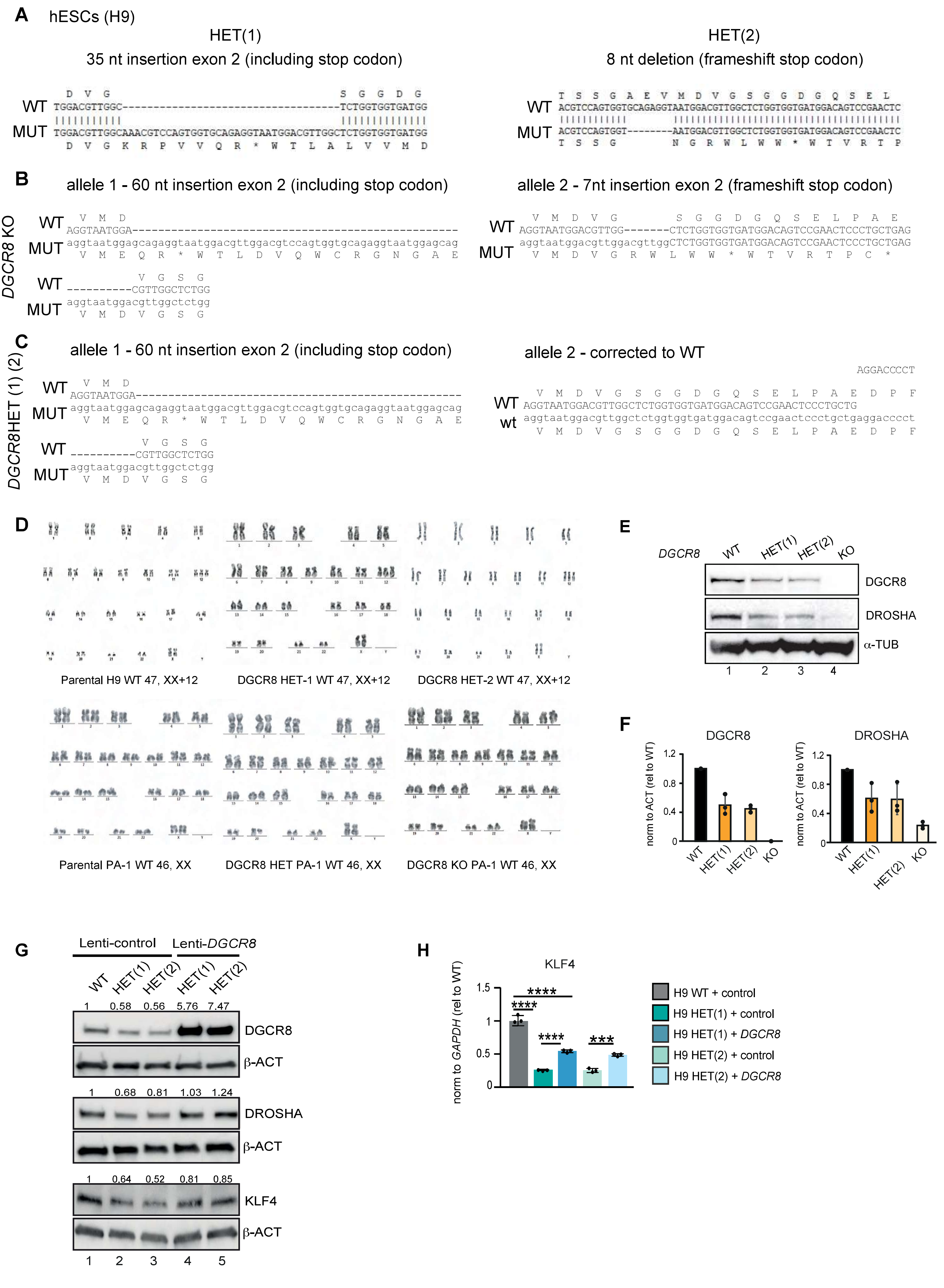

Supplementary Figure S1. Characterization of *DGCR8* heterozygous human pluripotent cell models.

(**A**) Sanger sequencing of H9 hESC *DGCR8* HET clone 1 (left) and clone 2 (right). HET(1) contains a 35-nt insertion in one allele of *DGCR8* that contains a stop codon. HET(2) contains a frameshift 8-nt deletion in one allele of *DGCR8* (**B**) Sanger sequencing of *DGCR8* KO PA-1 cells, allele 1 contains a 60 nt insertion which includes a stop codon, and allele 2 contains a frameshift 7-nt insertion in exon 2 (**C**) Sanger sequencing of *DGCR8* PA-1 HET clones (both HET1 and HET2 are genetically identical). HET cells were generated by correcting the frameshift 7-nt insertion in exon 2 of *DGCR8* in KO cells (**D**) Karyotype analyses of parental hESCs (H9) WT cells and both HET clones (upper panel) and parental PA-1 WT cells and DGCR8 KO and HET PA-1 clones used (**E**) DGCR8 and DROSHA western blot analyses of WT, two different HET DGCR8 PA-1 and KO clones. α-Tubulin serves as a loading control (**F**) Quantification of DGCR8 and DROSHA protein levels in PA-1 WT, HET and KO cells. Data represent the average of three independent experiments +/- st.dev. α-Tubulin serves as a loading control (**G**) DGCR8, DROSHA and KLF4 western blot analyses of H9 WT and HET DGCR8 hESCs transduced with a control or a lentiviral vector expressing human *DGCR8*. Actin serve as loading control (**H**) RT-qPCR analyses of KLF4 for WT and HET hESCs transduced with control or lentiviral vector expressing human *DGCR8*. Data are normalised to *GAPDH* and relative to WT levels. Data are the average (n=3) +/- st. dev. (***) p-val ≤ 0.001, (****) p-val ≤ 0.0001, by one-way ANOVA followed by Tukey's multiple comparison test.

Supplementary Figure S2

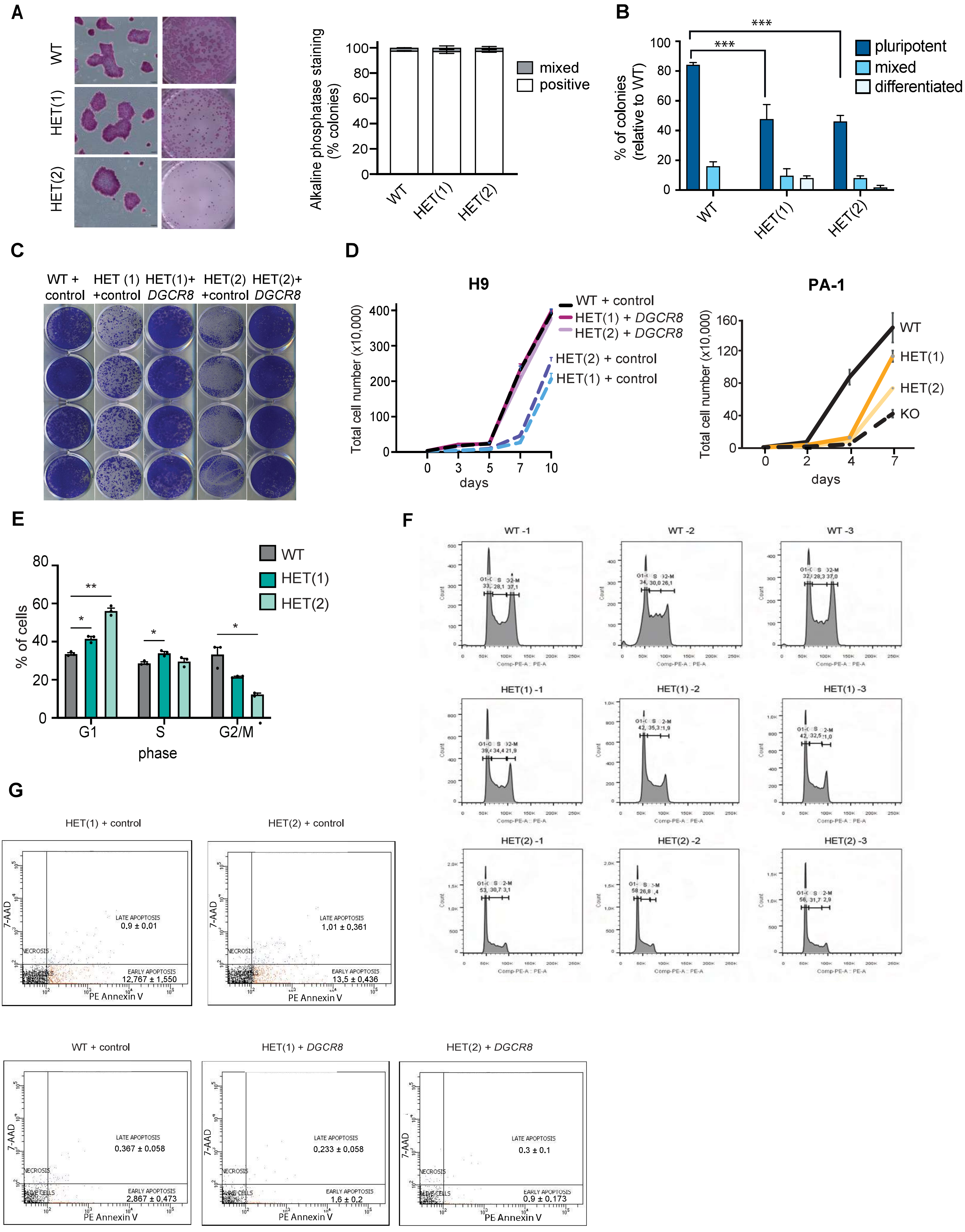

Supplementary Figure S2. *DGCR8* heterozygous human pluripotent cell models display proliferation and pluripotent defects.

(**A**) Alkaline phosphatase staining of parental cell line (WT) and HET H9 hESCs, with high (left) or low (right) magnification. Quantification of alkaline positive colonies, distinguishing between positive or mixed, regarding morphology and staining intensity (**B**) Quantification of alkaline positive, mixed or negative (differentiated) colonies, obtained after single-cell dilution of WT and HET cells. Data are the average of three independent experiments +/- st. dev., (***) p-val ≤ 0.001 by two-way ANOVA, followed by Dunnett's multiple comparison test (**C**) Clonal expansion assay for WT and HET cells transduced with a control or a lentiviral vector expressing human *DGCR8*. Colonies are visualised by crystal violet staining (**D**) Cell proliferation essay of WT and HET hESCs transduced with control and DGCR8-expressing lentiviruses (left) and PA-1 WT, HET and KO cells (right) (**E)** Cell cycle analyses by flow cytometry. Data represent the average of 3 biological replicates +/- st. dev. (*) p-val ≤ 0.05, (**) p-val ≤ 0.01 by two-way ANOVA followed by Dunnett's multiple comparison test (**F**) Cell cycle plots for three biological replicates are shown for WT and HET hESCs (**G**) Representative flow cytometry scatter plots for WT and HET hESCs transduced with a control lentiviral vector or DGCR8-expressing vector, stained with 7-AAD (y-axis) and PE Annexin V (x-axis). Cells positive for PE Annexin V are designated as 'early apoptosis', double labelled are considered 'late apoptosis'.

Supplementary Figure S3

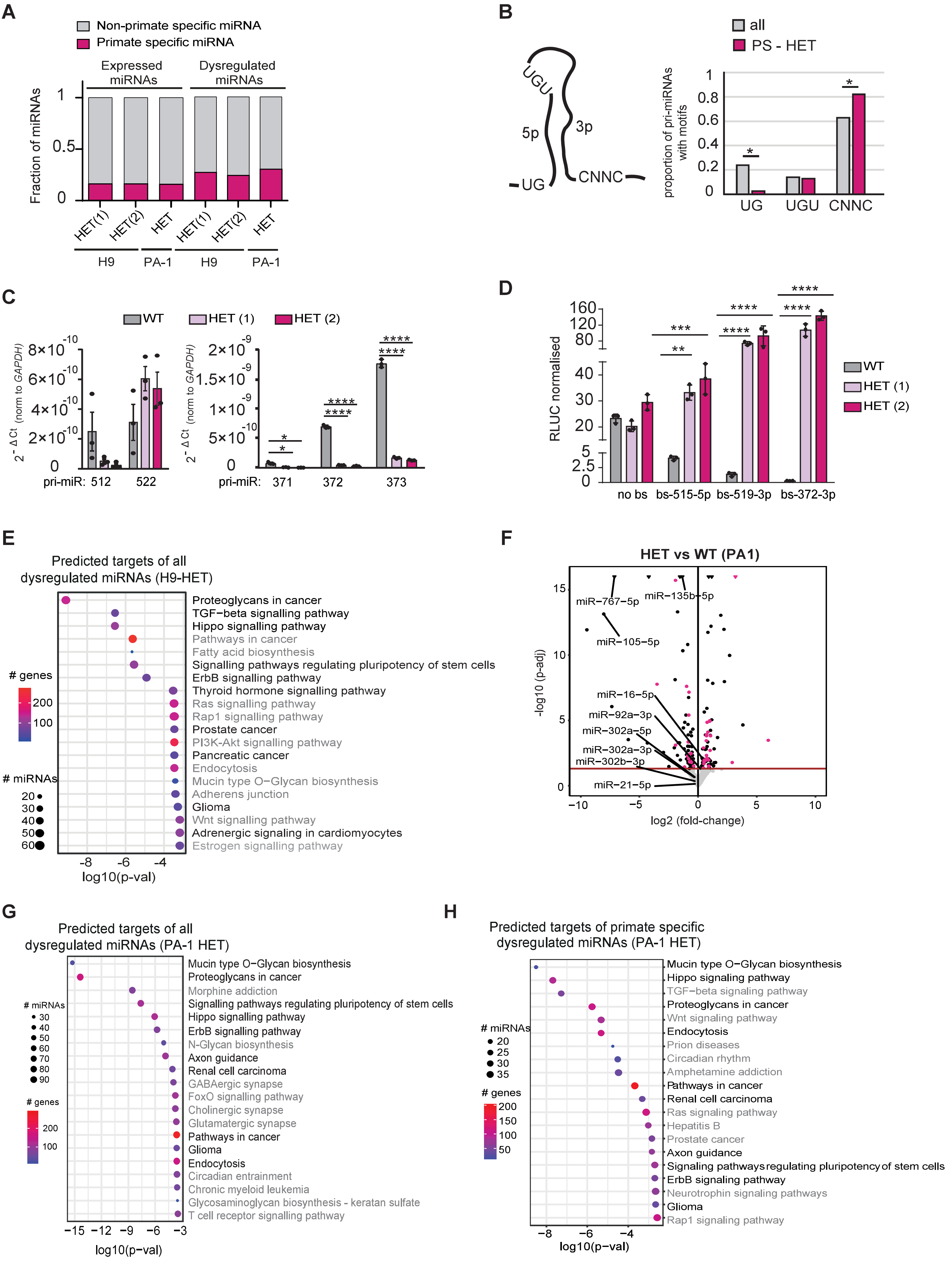

Supplementary Figure S3. Functional analysis of primate-specific dysregulated miRNAs in both human pluripotent cell models.

(**A**) Fraction of expressed and significantly dysregulated primate-specific miRNAs (pink). Dysregulated miRNAs were significantly enriched in primate-specific miRNAs in all HET cell lines: HET1 H9 hESCs (p-val =6.835985e-05), HET2 H9 hESCs (p-val =2.488492e-04) and HET PA-1 (p-val =3.65392e-07) (**B**) Relative frequency of UG, UGU and CNNC motifs was calculated for all annotated human pri-miRNAs (MiRGeneDB 3.0) versus the frequency found in primate-specific miRNAs dysregulated in HET hESCs. P-value was calculated using the Chi-square test (UG p-val = 0.0151; CNNC p-val = 0.0203) (**C**) Quantification of unprocessed pri-miRNA levels by RT-qPCR in WT and HET hESCs. Left panel and right panel represent pri-miRNAs belonging to C19MC and miR-371-373 clusters, respectively. Data are normalised to *GAPDH.* Data are the average (n=3) +/- st. dev. (*) p-val ≤ 0.5, (****) p-val ≤ 0.0001 by two-way ANOVA followed by Tukey's multiple comparison test (**D**) WT and HET hESCs were transfected with different psiCHECK2 constructs: empty vector as no binding site control "no bs" and psiCHECK containing perfect binding sites for miR-515-5p, miR-519c-3p or miR-572-3p clones in the 3'UTR of Rluc gene. Renilla luciferase measurements were normalized to the activity of firefly luciferase. Data are presented as RLU (Relative Luminescence Units) normalized, mean values ± st. dev. of three biological replicates. Statistically significant (**) p-val ≤ 0.01, (***) p-val ≤ 0.001 (****), p-val ≤ 0.0001 was calculated as two-way ANOVA followed by Tukey's multiple comparison test (**E**) KEGG pathway analyses for the predicted targets (microT-CDS) of all the common significantly dysregulated miRNAs in HET DGCR8 hESCs (**F**) Volcano plot of differential miRNA expression in HET PA-1 cells vs WT. MiRNAs in pink are primate-specific miRNAs (**G**) KEGG pathway analyses for predicted targets (microT-CDS) of dysregulated miRNAs

(TOP100) in HET PA-1 cells (**H**) the same as (**G**), but only using primate-specific miRNA predicted targets. Common pathways in (**G**) and (**H**) are in black, unique in grey.

Supplementary Figure S4

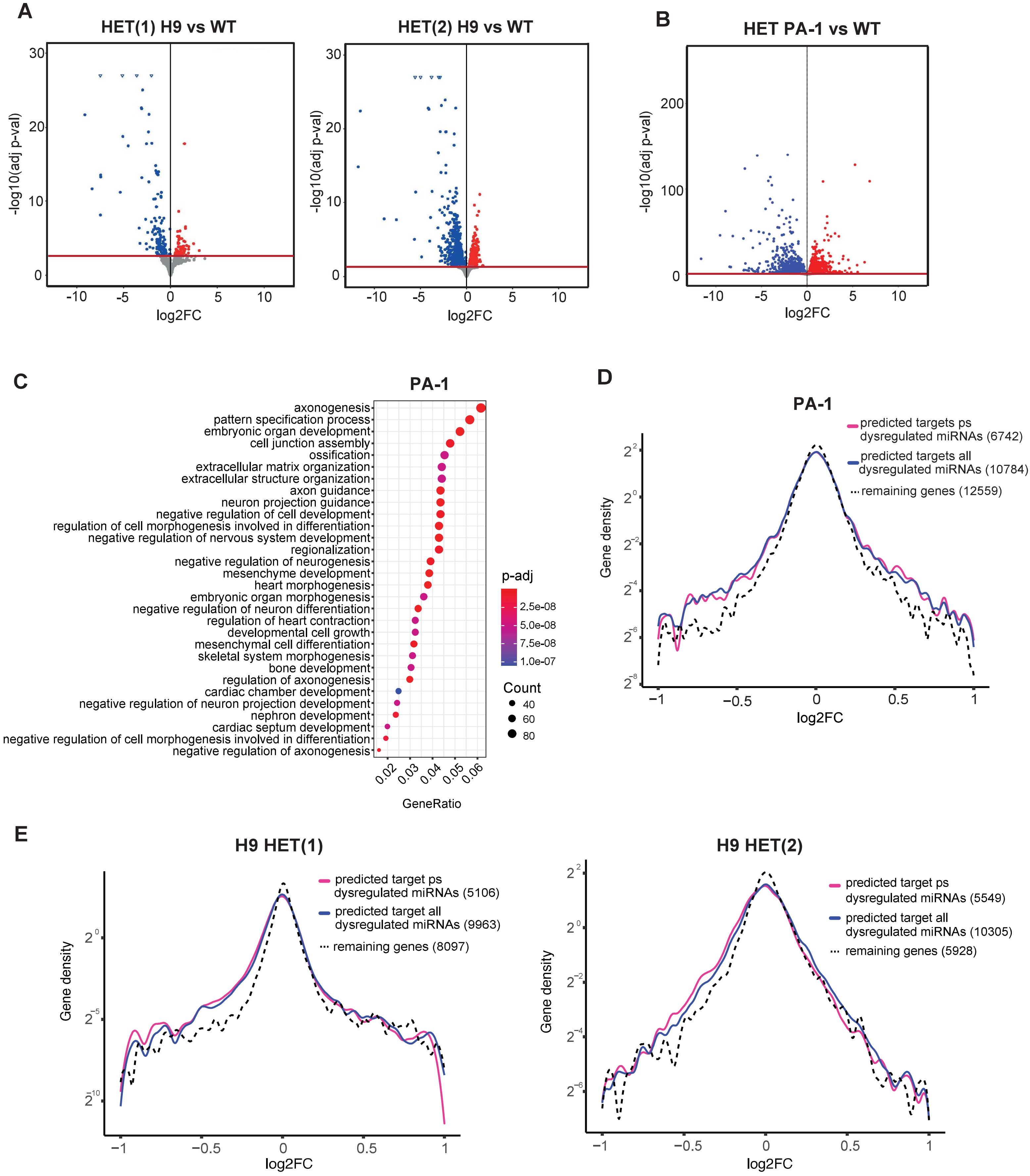

Supplementary Figure S4. Analysis of gene expression defects in both human pluripotent cell models.

(**A**) Volcano plots for differentially expressed genes in the two HET hESC clones vs WT hESCs. Blue are downregulated genes, and red, upregulated (**B**) the same as in (**A**) for HET PA-1 cells (**C**) GO pathway enrichment for differentially expressed genes in *DGCR8* HET PA-1 cells (only included TOP 30 categories) (**D-E**) Kernel density estimation of log2FC distributions for predicted targets of all dysregulated miRNAs (blue), targets of primate-specific dysregulated miRNAs (pink) and controls or non-target genes (black) for (**D**) PA-1 HET cells and (**E**) HET (1) and HET (2) hESCs. Distribution is significantly different for both targets of all dysregulated miRNAs and primate-specific miRNAs vs. non-targets (p-val $< 2.22e-16$).

Supplementary Figure S5

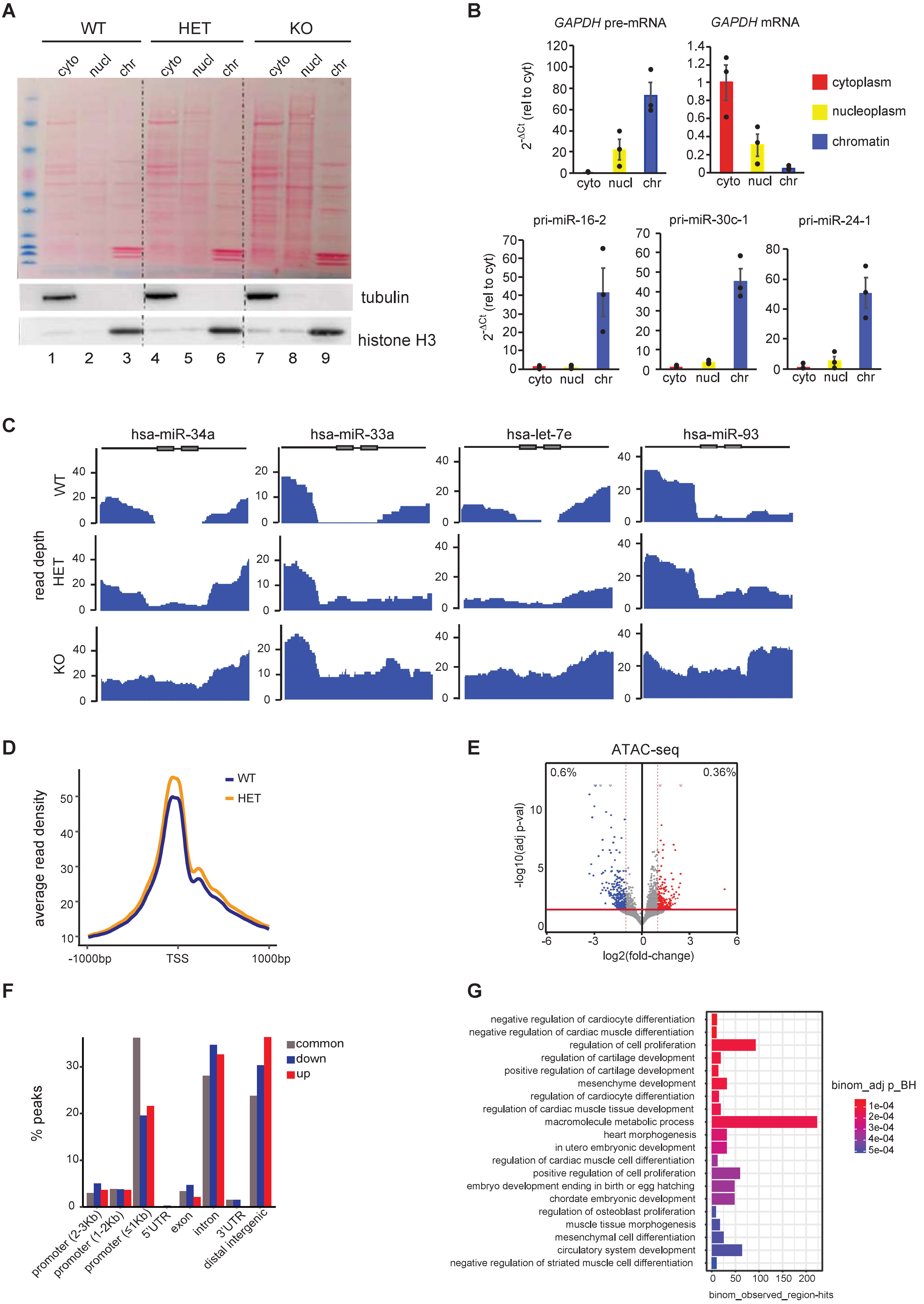

Supplementary Figure S5. *In vitro* pri-miRNA processing, chromatin-associated RNA enrichment and ATAC-seq analysis.

(**A**) Top, Ponceau staining of cytoplasmic (cyto), nucleoplasmic (nucl) and chromatin (chr) fractions from WT, HET and KO PA-1 cells. Bottom, western blot analyses of the same fractions against TUBULIN, which serves as a cytoplasmic fraction marker, and histone H3, which is a chromatin marker (**B**) RT-qPCR analysis of cytoplasmic, nuclear and chromatin fractions. *GAPDH* pre-mRNA serves as a positive control for chromatin fractions, while *GAPDH* mRNA is enriched in the cytoplasm. All three tested pri-miRNAs were enriched in the chromatin fractions from WT PA-1 cells. An equal proportional volume from the three fractions was used for cDNA preparation and qPCR analyses. Data for each primer pair are represented as a fold-change over the 'cytoplasmic' sample (**C**) Read depth coverage across several DGCR8-dependent miRNAs from chromatin-associated RNA high-throughput sequencing in WT, HET and KO PA-1 cells. Grey boxes indicate mature miRNAs, black line represents surrounding genomic regions (**D**) Read distribution around TSS (transcription start sites) of ATAC-seq peaks for WT (blue), and HET (orange) PA-1 cell lines (**E**) Differential ATAC accessibility analysis using DEseq2 of HET vs WT PA-1 cells (selected up/down hits with abs. log2FC ≥ 1, p-adj $\leq$ 0.05, hits outside plot as open triangles) (**F**) Proportion and genomic distribution of common (grey), less accessible (down, blue), or more accessible ATAC peaks (red, up) in HET vs WT PA- 1 (UTR; untranslated region) (**G**) Top twenty more significant GO terms associated with genomic regions that are significantly less accessible in HET cells obtained by rGREAT package.

Supplementary Figure S6

A

Predicted targets	miRNAs	Binding sites	Base Mean HET (1)	Base Mean HET (2)	Log2FoldChange HET (1)	Log2FoldChange HET (2)	p-adjust HET (1)	p-adjust HET (2)
ZNF398	Hsa-miR-515-5p	3x 6 mer	4557,22	4305,14	0,710	0,487	2,645E-02	2,360E-02
		1x 8mer						
HEY2	Hsa-miR-515-5p	7 mer	520,24	479,21	0,916	0,645	2,397E-03	1,984E-03
	Hsa-miR-519c-3p	8 mer						
		7 mer						

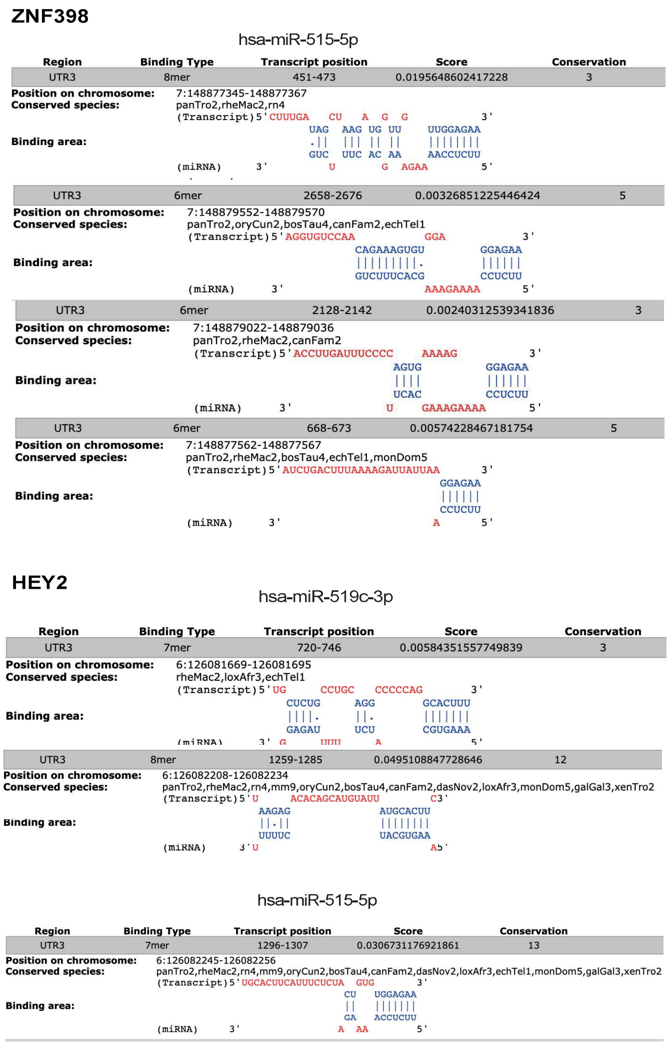

B

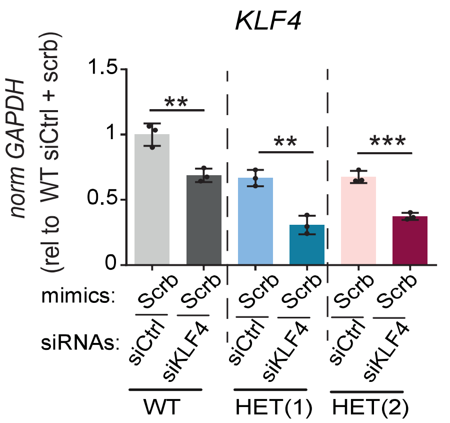

C

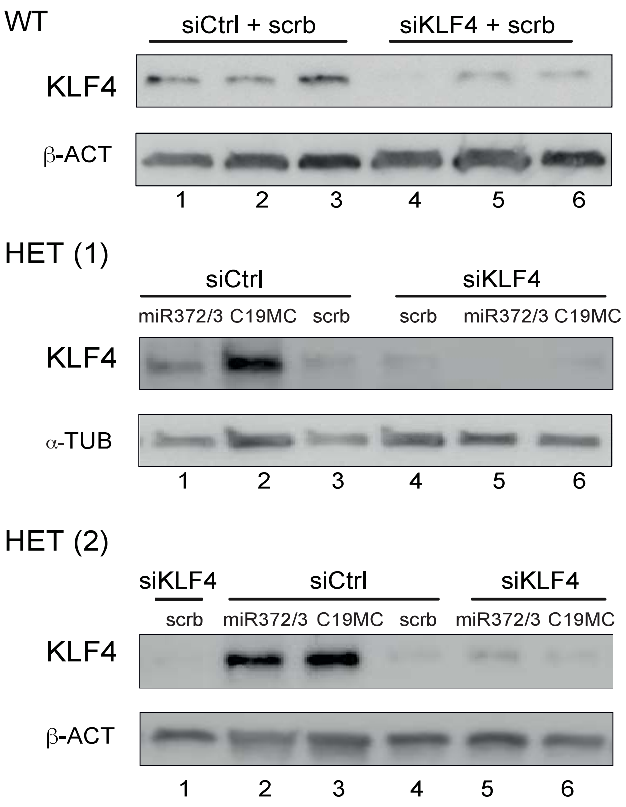

D

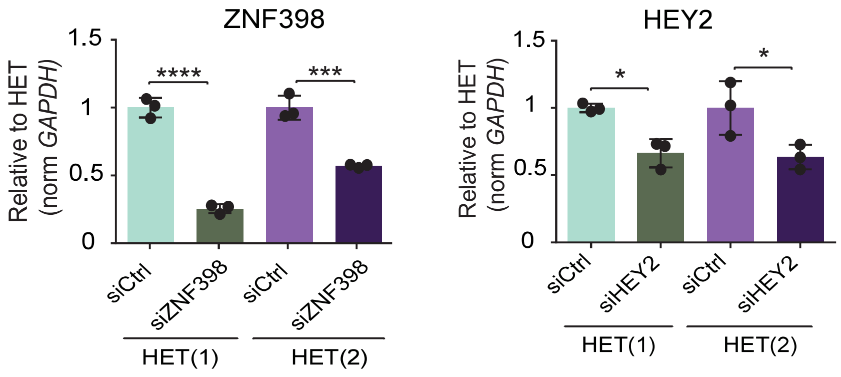

Supplementary Figure S6. Analysis of the mechanisms leading to the rescue of the molecular defects in HET hESC by the C19MC and miR-371-373 clusters

(**A**) *ZNF398* and *HEY2* genes are predicted targets of the hsa-miR-515-5p and hsa-miR-519c-3p. Number and type of miRNA binding sites, Base mean value, log2foldchange changes in expression and p-adjust value obtained by RNA-seq for each HET line are summarised in the table (top panel). Predicted hsa-miR-515-5p and hsa-miR-519c-3p binding sites in *ZNF398* and *HEY2* transcripts obtained from Diana tools are depicted at the bottom panel **(B)** RT-qPCR of *KLF4* in WT and HET hESCs co-transfected with siRNA control or siRNA against *KLF4* and miRNA control mimics (scrb). Data are normalised to *GAPDH* and relative to WT cotransfected with siRNA and miRNA mimic controls (scrb) . Data are the average (n=3) +/- st. dev. (**) p-val ≤ 0.01, (***) p-val ≤ 0.001, by two-tailed Student t-test (**C**) KLF4 western-blot analyses of WT and HET cells co-transfected with siRNAs (siCtrl or siKLF4) and miRNA mimics (Scrb control or the pool of mimics belonging to 371-3 cluster or C19MC cluster). ®-actin or α-tubulin serves as a loading control (**D**) RT-qPCR analyses of *ZNF398* and *HEY2* levels after transfection with siRNA control (siCtrl) or siRNA against *ZNF398* or *HEY2.* Data are normalised to *GAPDH* and relative to HET siCtrl levels. Data are the average (n=3) +/- st. dev. (*) p-val ≤ 0.5, (***) p-val ≤ 0.001 (****), p-val ≤ 0.0001, by one-way ANOVA, followed by Tukey's multiple comparison test.

Supplementary Figure S7

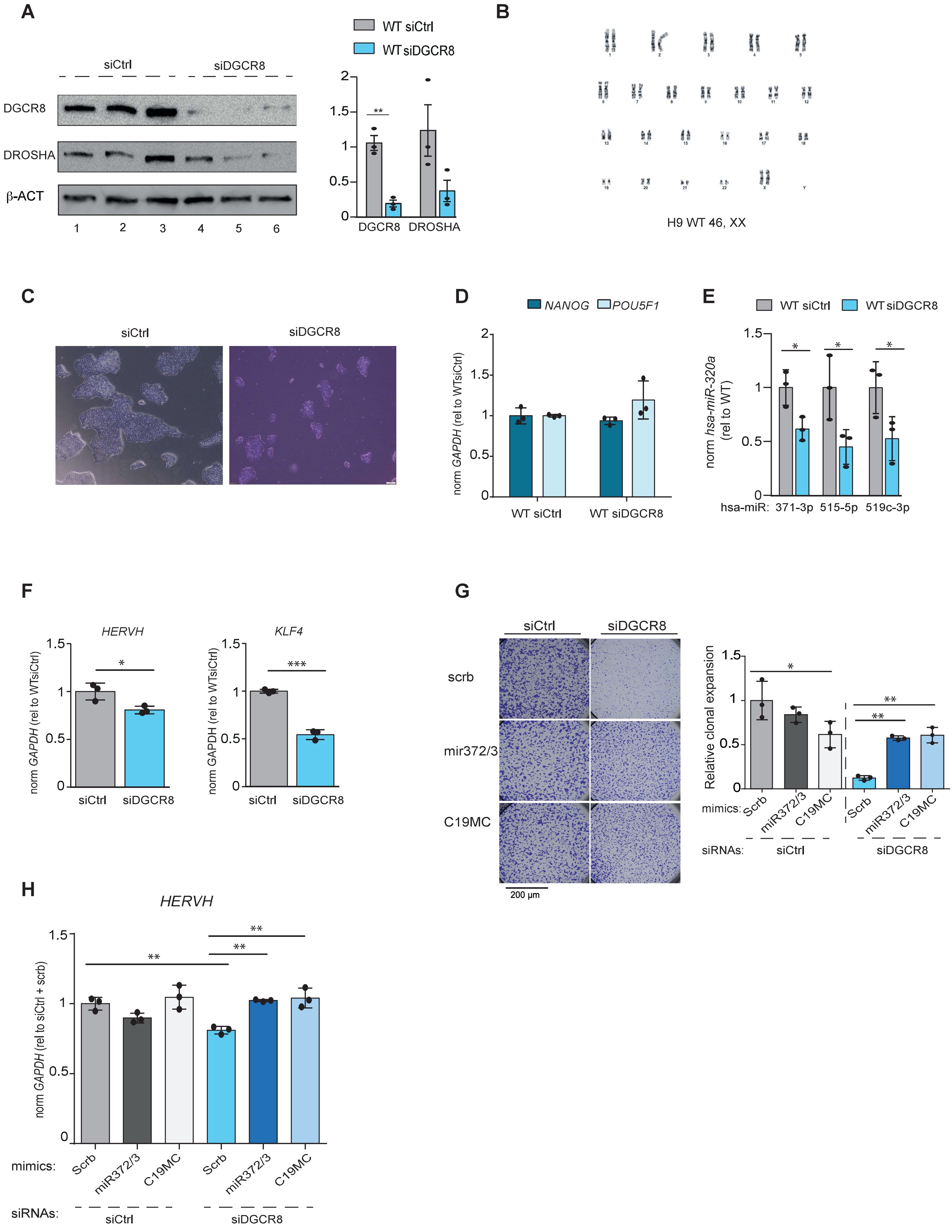

Supplementary Figure S7. Transient *DGCR8* depletion in HET hESCs results in similar molecular and cellular defects.

(**A**) DGCR8 and DROSHA western blot analyses of WT H9 hESCs after transfection with siRNA control (siCtrl) or siRNA against *DGCR8 (*siDGCR8*)*. ®-actin serves as a loading control (**B**) Karyotype analyses of the hESCs used (**C**) Colony morphology in normal culturing conditions for hESCs 6 days after transfection with siCtrl or siDGCR8 (Scale bar = 100 μm) (**D**) RT-qPCR for pluripotency markers *NANOG* and *POU5F1*, 24 hours after the second round of siRNA depletion. Data represent the average of three biological replicates +/- st. dev. No significant changes after one-way ANOVA analysis, followed by Tukey's multiple comparison test (**E**) RT-qPCR of mature *hsa-miR-371-3p*, hsa-*miR-515-5p* and *hsa-miR-519c-3p* expression in hESCs after DGCR8 depletion with siRNAs. Data represent the average (n=3) +/- st. dev. Expression levels for each miRNAs are normalised to the levels of the DGCR8-independent miRNA, *hsa-miR-320-3p,* and expressed relative to levels in the non-targeting siRNA control (siCtrl), (*) p-val ≤ 0.05, by one-way ANOVA followed by Dunnett's multiple comparison test (**F**) Quantification of *HERVH* and *KLF4* levels by RT-qPCR after depletion of DGCR8 (siDGCR8) or using a siRNA control (siCtrl). Data are the average (n=3) +/- st. dev. (*) p-val ≤ 0.5, (***) p-val ≤ 0.001, by two-tailed Student t-test (**G**) Relative clonal expansion capacity of hESC co-transfected with a siRNA (control or against *DGCR8*) plus miRNA mimics (Scrb control or the pool of mimics belonging to 371-3 cluster or C19MC cluster). Relative clonal expansion quantification is expressed as the stained area and normalized to hESCs transfected with siCtrl and Scrb mimic. Data represents the average +/- st. dev. of 3 biological replicates. (*) p-val ≤ 0.5, (**) p-val ≤ 0.01, by one-way ANOVA followed by Tukey's multiple comparison test (right). Representative images of colonies stained after 6 days in culture (left) (Scale bar = 200 μm) (**H**) Quantification of HERVH levels

by RT-qPCR after transfection with a siRNAs against *DGCR8* and miRNA mimics (Scrb control or the pool of mimics belonging to 371-3 cluster or C19MC cluster). Data represent the average of three biological replicates +/- st. dev. (**) p-val ≤ 0.01, by one-way ANOVA followed by Tukey's multiple comparison test. Data are normalised to *GAPDH* and represented relative to hESCs controls transfected with non-targeting siRNA (siCtrl) and scrambled non-targeting mimics (Scrb).

Supplementary Figure S8

Uncropped Western blots membranes used for Figure 1B, 3B and Supplementary Figure S1G

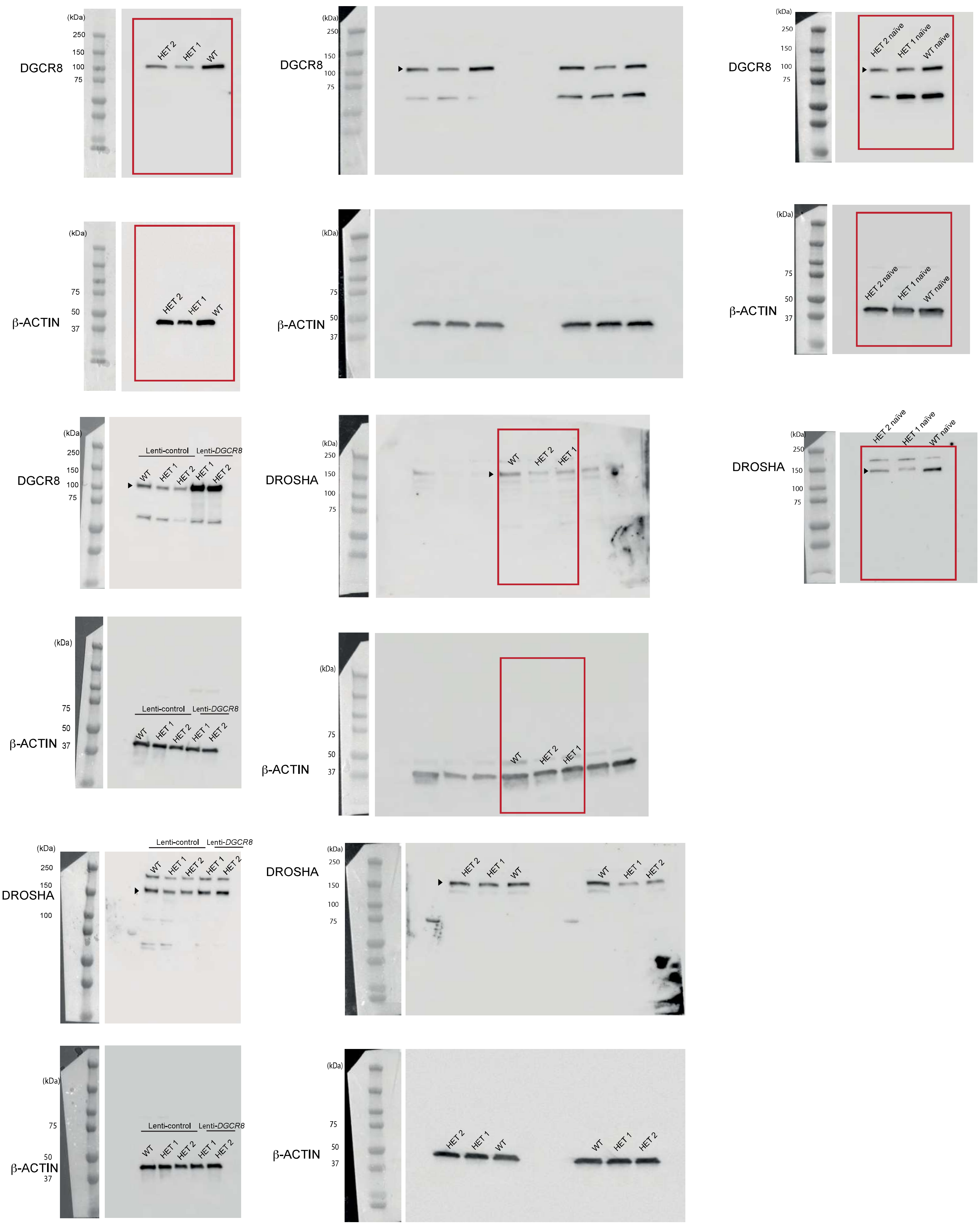

Supplementary Figure S9

Uncropped Western blots membranes used for Supplementary Figure S1E

PA-1 DGCR8 Nterm

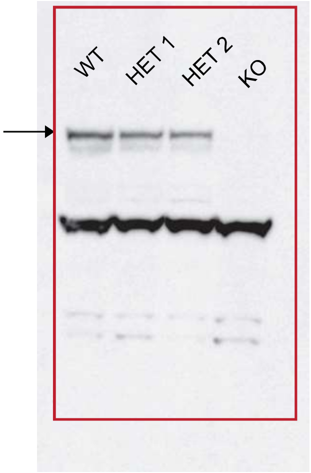

PA-1 Drosha

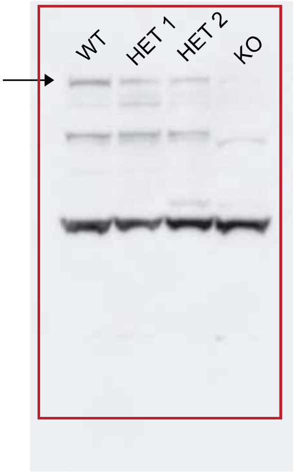

PA-1 α-tubulin

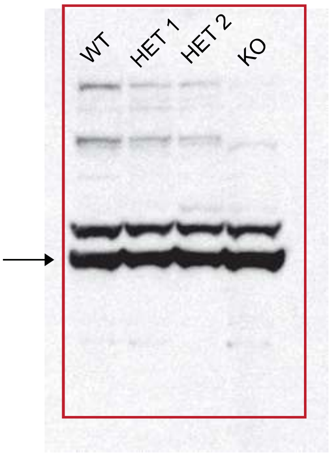

PA-1 DGCR8 Nterm

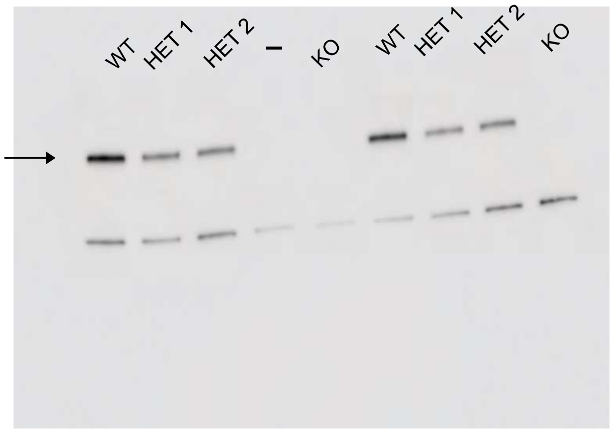

PA-1 Drosha

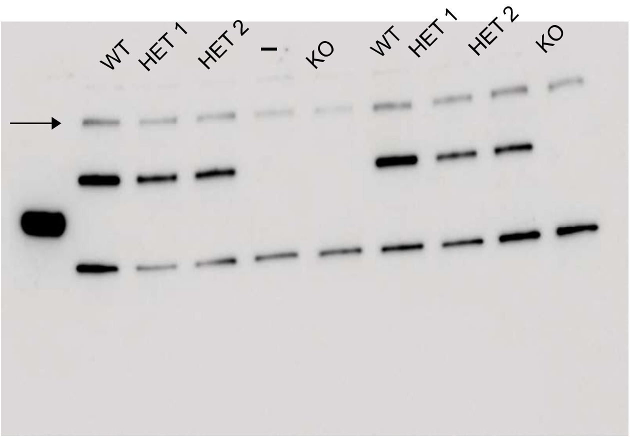

PA-1 actin

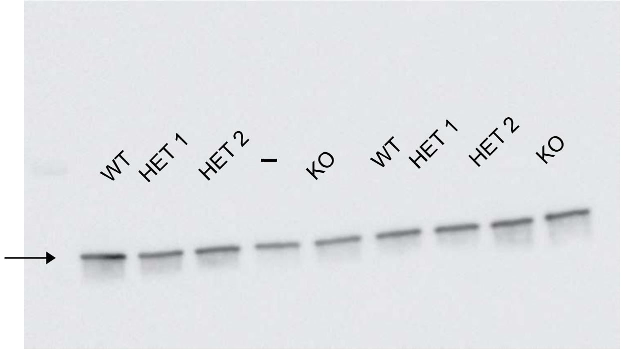

Supplementary Figure S10

Uncropped Western blots membranes used for Figure 1E and Supplementary Figure S1G

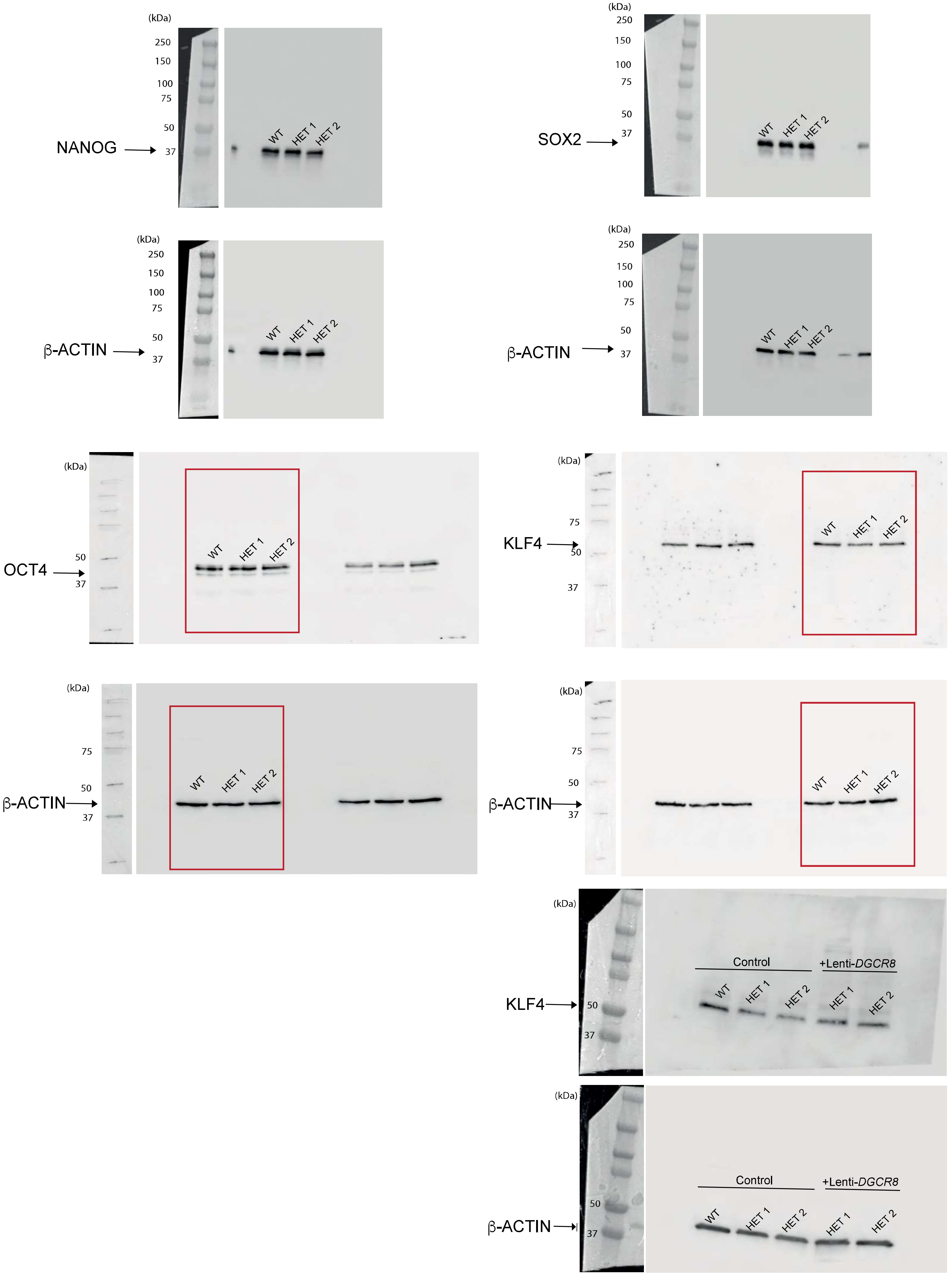

Supplementary Figure S11

Uncropped Western blots membranes used for Figure 7B and 7F

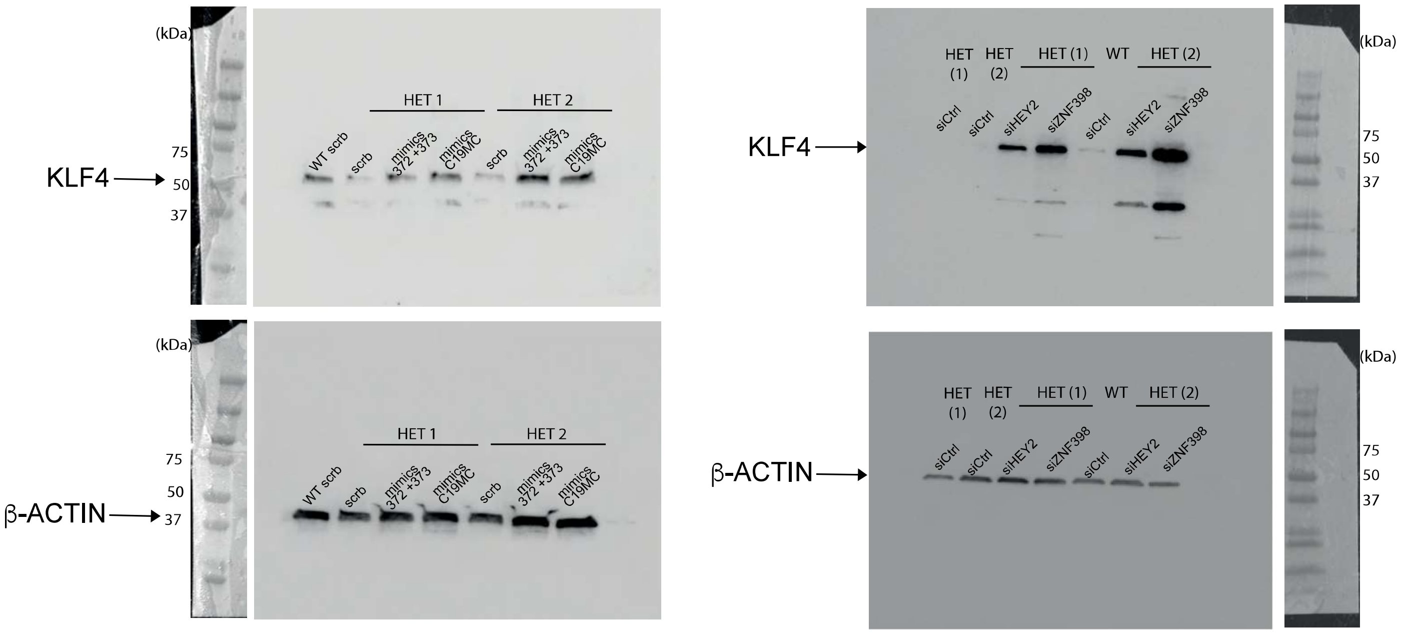

Supplementary Figure S12

Uncropped Western blots membranes used for Supplementary Figure S6C and S7A

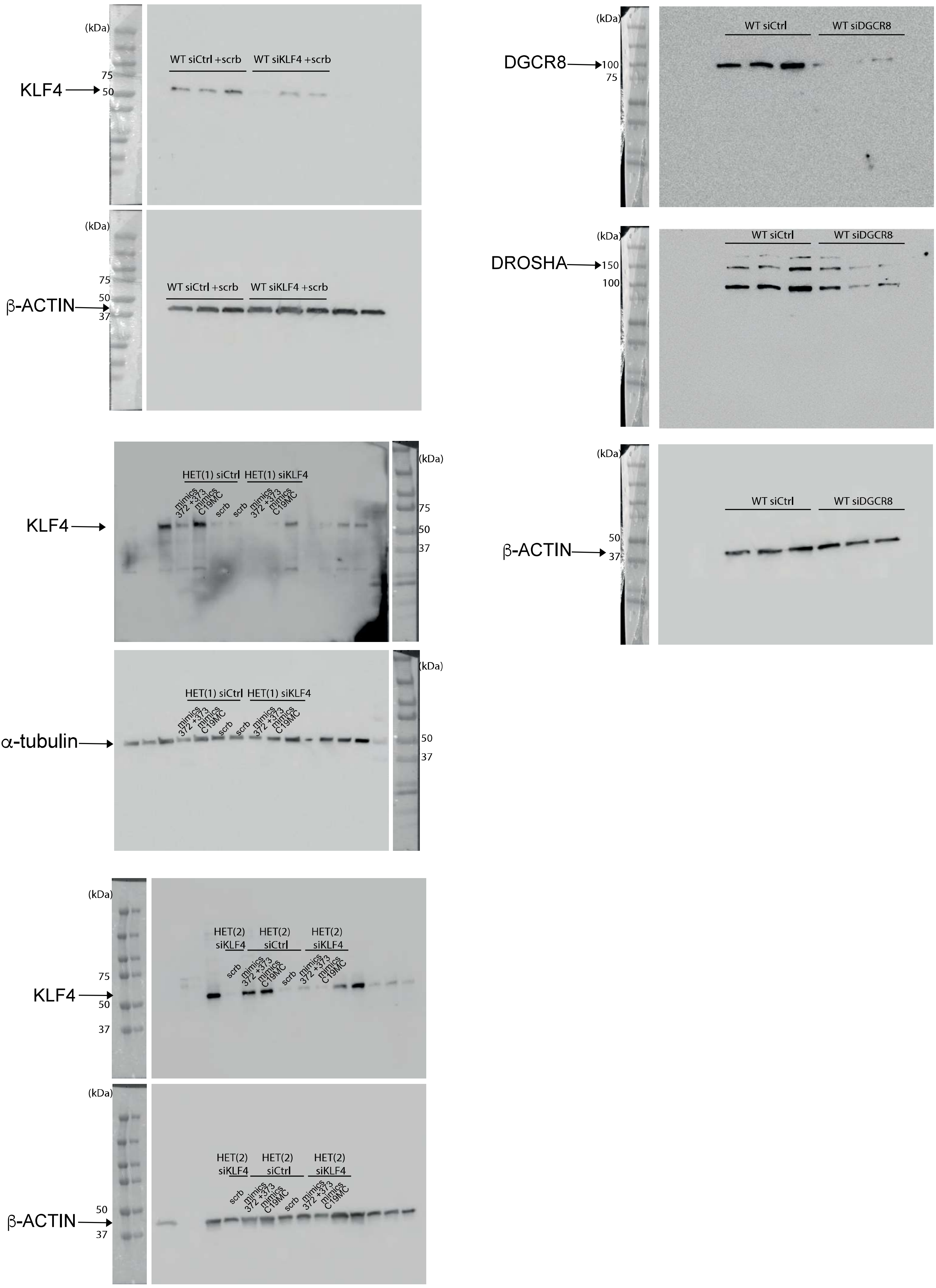

Supplement: Supplementry Figures [file EMS204119-supplement-Supplementry_Figures.pdf]
